# Network-Level Structural Abnormalities of Cerebral Cortex in Type 1 Diabetes Mellitus

**DOI:** 10.1371/journal.pone.0071304

**Published:** 2013-08-23

**Authors:** In Kyoon Lyoo, Sujung Yoon, Perry F. Renshaw, Jaeuk Hwang, Sujin Bae, Gail Musen, Jieun E. Kim, Nicolas Bolo, Hyeonseok S. Jeong, Donald C. Simonson, Sun Hea Lee, Katie Weinger, Jiyoung J. Jung, Christopher M. Ryan, Yera Choi, Alan M. Jacobson

**Affiliations:** 1 Ewha Brain Institute & College of Pharmacy, Graduate School of Pharmaceutical Sciences, Ewha W. University, Seoul, South Korea; 2 Department of Psychiatry, Catholic University of Korea College of Medicine, Seoul, South Korea; 3 The Brain Institute, The University of Utah, Salt Lake City, Utah, United States of America; 4 Department of Psychiatry, Soonchunhyang University College of Medicine, Seoul, South Korea; 5 Research Division, Joslin Diabetes Center, Boston, Massachusetts, United States of America; 6 Department of Psychiatry, Harvard Medical School, Boston, Massachusetts, United States of America; 7 Department of Brain and Cognitive Sciences, Ewha W. University Graduate School, Seoul, South Korea; 8 Interdisciplinary Program in Brain Science, Seoul National University College of Natural Sciences, Seoul, South Korea; 9 Department of Internal Medicine, Brigham and Women’s Hospital, Boston, Massachusetts, United States of America; 10 Department of Psychiatry, University of Pittsburgh School of Medicine, Pittsburgh, Pennsylvania, United States of America; 11 Department of Psychiatry, Winthrop University Hospital, Mineola, New York, United States of America; Institute of Psychology, Chinese Academy of Sciences, China

## Abstract

Type 1 diabetes mellitus (T1DM) usually begins in childhood and adolescence and causes lifelong damage to several major organs including the brain. Despite increasing evidence of T1DM-induced structural deficits in cortical regions implicated in higher cognitive and emotional functions, little is known whether and how the structural connectivity between these regions is altered in the T1DM brain. Using inter-regional covariance of cortical thickness measurements from high-resolution T1-weighted magnetic resonance data, we examined the topological organizations of cortical structural networks in 81 T1DM patients and 38 healthy subjects. We found a relative absence of hierarchically high-level hubs in the prefrontal lobe of T1DM patients, which suggests ineffective top-down control of the prefrontal cortex in T1DM. Furthermore, inter-network connections between the strategic/executive control system and systems subserving other cortical functions including language and mnemonic/emotional processing were also less integrated in T1DM patients than in healthy individuals. The current results provide structural evidence for T1DM-related dysfunctional cortical organization, which specifically underlie the top-down cognitive control of language, memory, and emotion.

## Introduction

The central nervous system (CNS) serves as one of the representative end-organ targets of metabolic insult related to type 1 diabetes mellitus (T1DM) [1]. End-organ damage to the brain by T1DM involves changes in synaptic plasticity as well as direct damage to neurons [1]. Given the dynamic nature of the brain regional organization in response to neuronal damage [2]–[4], disease processes of diabetes may elicit rearrangement of the intra- and inter-regional brain architectures. Furthermore, because T1DM may begin in early childhood [5] when the brain undergoes profound developmental changes such as neuronal proliferation, synaptic pruning, and myelination [6]–[8], the potential for brain structural and functional reorganization may increase in response to metabolic insult by T1DM.

To render the human brain capable of exerting its characteristic functions, it is essential not only that individual neuroanatomical areas responsible for specific function are operational but also that the complex and hierarchal networks between corresponding areas are comparably efficient [9]. Recent advances in image analysis techniques have enabled us to identify and evaluate the complex organization of brain regional connections using graph theoretical network analysis [9], [10]. Specifically, structural network analysis using interregional covariance of cortical thickness measurements can provide important information on structural interactive connectivity of the human brain [11]–[14]. Disrupted structural network organization has increasingly been reported in aged population [14], as well as in patients with schizophrenia [15], dementia [16], [17], epilepsy [18], and multiple sclerosis [13]. However, to the best of our knowledge, no studies thus far have examined the inter-regional network efficiencies of the T1DM brain, despite well-recognized T1DM-related end-organ damages to the brain [1]. In the present study, we sought to determine the differences in topological attributes of underlying cortical structural networks between young adults with T1DM and healthy adults. To investigate broad network architectures with emphasis on their functional significance, we divided the cerebral cortex into the intrinsic structural sub-network systems that mainly subserve cortical functions including strategic/executive control, language, mnemonic/emotional processing, sensorimotor, and visual functions, based on prior knowledge of structural and functional modularity of regional connectivity [11], [19]. In light of the crucial role of the prefrontal regions in top-down cognitive control of other brain functions including language, mnemonic/emotional processing, and sensorimotor function [20]–[23], we expected reduced inter-network efficiencies between intrinsic structural sub-network systems in T1DM patients relative to control subjects.

## Materials and Methods

### Ethics statement

The study protocol was approved by the institutional committee on human subjects of the Joslin Diabetes Center, Boston, MA, USA, the Brain Imaging Center of the McLean Hospital, Boston, MA, USA, and the Seoul National University Hospital, Seoul, South Korea. All participants were given a complete explanation of the study details and then provided written informed consent to participate in the study.

### Participants

Adult T1DM patients between the ages of 25 and 40 and age- and sex-matched healthy control subjects were recruited from the Joslin Diabetes Center. The sample has been described in detail elsewhere [24]. Among subjects of the original cohort, 81 T1DM patients and 38 healthy subjects who had never experienced a depressive episode were included in the present study.

Acquisition of high-resolution of MR images was performed at the Brain Imaging Center of the McLean Hospital. Subjects were excluded if they had major neurological or medical disorders, if they had ever been diagnosed with psychosis, schizophrenia, bipolar disorder, attention-deficit hyperactivity disorder, or cocaine, heroin, or alcohol dependence, as assessed using the Structural Clinical Interview for DSM-IV [25], or if they had any contraindications to magnetic resonance (MR) imaging. Serious diabetic complications including clinically significant diabetic nephropathy, painful or symptomatic neuropathy, proliferative retinopathy requiring laser treatment, or gastroparesis were additional exclusion criteria for the T1DM group. Diabetic characteristics were obtained from the medical records and laboratory tests. Lifetime average hemoglobin A1C (HbA1C) was measured using the average value of HbA1C levels, grouped and time-weighted every 4 years for entire duration of the disease [24], which indicates the level of lifetime glycemic control. Among 57 T1DM patients whose information regarding insulin therapy is available, 39 T1DM patients received rapid- or short-acting insulin analogues while 33 did intermediate- or long-acting insulin analogues. Several patients were treated with one or more classes of insulin analogues. Twelve patients were managed using the insulin pump. Demographic and clinical characteristics of study participants are demonstrated in Table 1.

**Table 1:** Group characteristics of patients with T1DM and control subjects.

Characteristics	T1DM patients (n = 81)	Control subjects (n = 38)	*P* Value a
**Demographics**			
Mean age (SD), *y*	32.5 (4.6)	30.8 (5.1)	0.08
Female, *n (%)*	41 (50.6)	19 (50.0)	0.95
Right handedness, *n (%)*	79 (97.5)	35 (92.1)	0.33
**Diabetes–specific clinical characteristics**			
Duration of illness (SD), *y*	19.8 (3.5)	NA	NA
Onset age (SD), *y*	12.8 (5.1)	NA	NA
Lifetime average HbA1C (SD) b, *%*	7.99 (1.19)	NA	NA
No. of hypoglycemic episodes (SD) c	6.41 (14.7)	NA	NA
Current HbA1C (SD), *%*	7.73 (1.43)	5.08 (0.33)	<0.001

a Group differences were tested by independent t-tests or χ^2^ tests appropriately.

b Average value of HbA1C, grouped and time-weighted every 4 years for the duration of illness.

c A severe hypoglycemic episode was defined as an event that leads to a coma, seizure, or unconsciousness according to the Diabetes Control and Complications Trial Research Group Criteria.

Abbreviations: T1DM, type 1 diabetes mellitus; SD, standard deviation; HbA1C, hemoglobin A1C; NA, not available or not applicable.

### MR image acquisition and measurement of cortical thickness

Brain MR images were obtained using a 1.5 Tesla GE whole body imaging system (Horizon LX, GE Medical systems, Milwaukee, Wisconsin, USA) at the Brain Imaging Center of the McLean Hospital. Coronal T1 weighted images were produced using a three–dimensional spoiled gradient echo pulse sequence (124 slices, slice thickness  = 1.5 mm, echo time [TE]  = 5 ms, repetition time [TR]  = 35 ms, matrix  = 256×192; field of view [FOV]  = 24 cm, flip angle [FA]  = 45°, number of excitations [NEX]  = 1). Axial T2 weighted images (TE  = 80 ms, TR = 3,000 ms, 256×192 matrix; FOV  = 20 cm, FA = 90°, NEX  = 0.5, slice thickness/gap  = 3/0 mm) and Fluid Attenuated Inversion Recovery axial images (TE  = 133 ms, TR  = 9,000 ms, inversion time  = 2,200 ms, matrix  = 256×192, FOV  = 20 cm, FA = 90°, NEX  = 1, slice thickness/gap  = 5/2 mm) were obtained to screen for potential brain structural abnormalities.

Cortical thickness measurements were conducted using the FreeSurfer Tools (http://surfer.nmr.mgh.harvard.edu/) and the method implemented in this study has been previously described in detail elsewhere [24], [26]–[28]. In brief, initial surface was obtained for further reconstructing the pial surface using deformable surface algorithm [29] after the segmentation of white matter [30], tessellation of gray/white matter boundaries [30], and automatic correction of topological defects [31]. All surfaces of each individual image data were visually inspected for accuracy of spatial registration and gray/white matter segmentation and were manually corrected, as recommended by the guideline. Cortical thickness at each vertex was defined as the shortest distances from the pial to the corresponding gray/white surface. This measurement for the thickness of the cerebral cortex has been validated using histological [32] and manual [32], [33] estimations with sub-millimeter precision. Reliability for this measurement has also been established across field strength, scanner upgrade, and manufacturer [34].

### Inter-regional correlations and construction of structural cortical networks

Cerebral cortices on MR images were parcellated into 32 gyral-based regions for each hemisphere, using an automated labeling system that is distributed in the FreeSurfer Tool (Figure S1 in File S1) [35]. Cortical thickness of each vertex belonging to the specific parcellated region was averaged to define cortical thickness of that region.

Parcellated regions were further categorized into five segregated intrinsic structural network systems subserving different neurobehavioral functions including strategic/executive control, language, mnemonic/emotional processing, sensorimotor, and visual functions based on prior shared knowledge on structural and functional clusters (Figure S1 in File S1) [11], [19]. The structural network system responsible for strategic/executive control was composed of 14 parcellated regions mainly located in the dorsolateral prefrontal and cingulate cortices. The structural network system subserving speech and language-related function comprised 14 parcellated regions mainly located in the auditory cortex as well as Broca's *and* Wernicke's areas. The structural network system supporting mnemonic/emotional processing was composed of 18 parcellated regions that are primarily located in medial prefrontal and paralimbic cortices**.** The structural network system responsible for sensorimotor function was composed of 10 parcellated regions primarily placed in the primary or association cortices for sensory or motor function. The structural network system supporting visual function was composed of 8 parcellated regions which are located in the occipital cortex.

Partial correlation analyses of cortical thickness in each possible pair of 64 parcellated regions after adjusting for age and sex were used for the measurement of structural associations between regions. Interregional connection matrices were made for each of the T1DM and control groups by calculating the partial correlation coefficients for all subjects in each study group, representing the specific anatomical connections between each of the 2016 [ = (64×63)/2] possible pairs of parcellated regions. Each connection matrix was thresholded into a binarized matrix with a fixed sparsity (*S*), which was defined as the total number of edges (connections) in the graph divided by the maximum possible number of edges, 2016 [14], [16]. This thresholding method enabled us to exclusively examine the alterations of topological organization based on connection matrices comprising same number of edges in each group [16], [36], [37]. A wide range of sparsity levels (0.07≤*S*≤0.40) were applied to threshold the connection matrix with an incremental interval of 0.01 and the topological organizations of resultant matrices at each sparsity level were examined for both study groups [14], [16]. Brain network analyses were performed with an open source Matlab toolbox (http://www.brain-connectivity-toolbox.net) [10].

### Measurement of global and local efficiencies of whole-brain structural cortical networks

Segregation and integration of whole-brain structural network reflect the efficiency of connections within densely interconnected regions and throughout remote brain regions, respectively [9]. The local efficiency (*E_loc_*), a common measure of segregation of cortical network, specifically quantifies how efficiently the network transfers information at the clustering level [10]. The global efficiency (*E_glob_*), a common measure of integration of cortical network, quantifies how efficiently the network exchanges the information at the global level [10].

### Measurement of inter-network organizations of intrinsic structural network systems

The length of path reflecting potential routes of information between regions could represent an estimate for efficiency of network integration [10], [38]. The average shortest path length (*L*) between all pairs of nodes in the network has been commonly used to measure the efficiency of a network [10], [38]. Structural sub-network systems subserving strategic/executive control, language, mnemonic/emotional processing, and sensorimotor function, which are known to be related to T1DM pathology, were at the focus of the present study.

Based on evidence of the prefrontal regions' vital role in top-down control for other brain function [20]–[23], inter-network efficiencies between structural network system for strategic/executive control and other intrinsic network systems were also measured by calculating the average inverse shortest path length (*1/L*) with the connection submatrix for corresponding network systems: strategic/executive control and language, strategic/executive control and mnemonic/emotional processing, and strategic/executive control and sensorimotor function.

### Network hub identification and analysis of hierarchical organization of whole-brain structural network

Among various measures of centrality [10], we used two measures to identify the regional hubs of structural network: degree (*K*) and betweenness-centrality (*b*). The degree of a specific region (*i*), *K_i_*, is defined as the number of edges (connections) to other regions throughout the network [10], [12]. The betweenness centrality of a specific region (*i*), *b_i_*, is defined as the number of shortest paths between any two regions that pass this specific region [10]. For hub identification, degree and the betweenness-centrality values were calculated at a specific sparsity threshold of 0.23. This threshold level was selected to ensure that all cortical parcellated regions of both study groups were included in the brain network to minimize the number of false-positive connections [16]. The regions with the normalized betweenness-centrality (*B_i_*) greater than 1.5 *and* the degree higher than the mean degree of whole-brain networks are considered as regional hubs of structural network [14], [16], [39]. The hierarchical organization has a feature characterized by having many hubs with high degree (total connectivity) but low clustering (local connectivity). The clustering coefficient (*C*) of a specific region (*i*), *C_i_,* is defined as the ratio of the number of connections of that region with its nearest regions (neighbors) to the maximum number of possible connections in the network [10]. The hierarchical structure of the network was measured by the β coefficient of the power-law relationship between the clustering coefficient (*C*) and degree (*k*): *C = k* ^–β^ [15], [40], [41]. We further classified hubs with lower clustering, which have clustering coefficients lower than the mean values of whole-brain structural networks, as hubs at a hierarchically higher level [9], [15].

As supplementary analyses, modularity of the structural cortical network (*m*) was also examined [11]. The process of modularity optimization found the optimal community structure, which is a subdivision of the structural cortical network into the non-overlapping groups of nodes, for achieving the maximum network modularity. We also defined intra-module connectivity of each node as the z score of the total number of degrees of a specific node (*i*) within a module (*Z_i_*) [42], [43]. Inter-modular connectivity was measured by the participation coefficient (*P_i_*) [42], [43].

### Statistical analyses

Group differences in demographic characteristics were analyzed using the independent t-test or chi-square test.

Differences in topological parameters for structural cortical network between the T1DM and control groups were examined using a non-parametric permutation test [44]. The regional cortical thickness values for each subject were randomly assigned to one of two groups and the partial correlation matrices for each randomized group were recomputed. Binarized matrices were then obtained using the same sparsity threshold as in the real brain network. The network parameters were calculated at each sparsity and between-group differences in all parameters were also obtained. The randomization steps were repeated 1000 times to sample the permutation distribution of differences in network parameters and 95% points of each distribution were used as critical values for a one-tailed test of null hypothesis with a probability of type 1 error of 0.05.

## Results

### Global and local efficiencies of whole-brain structural networks

**Figure 1:**
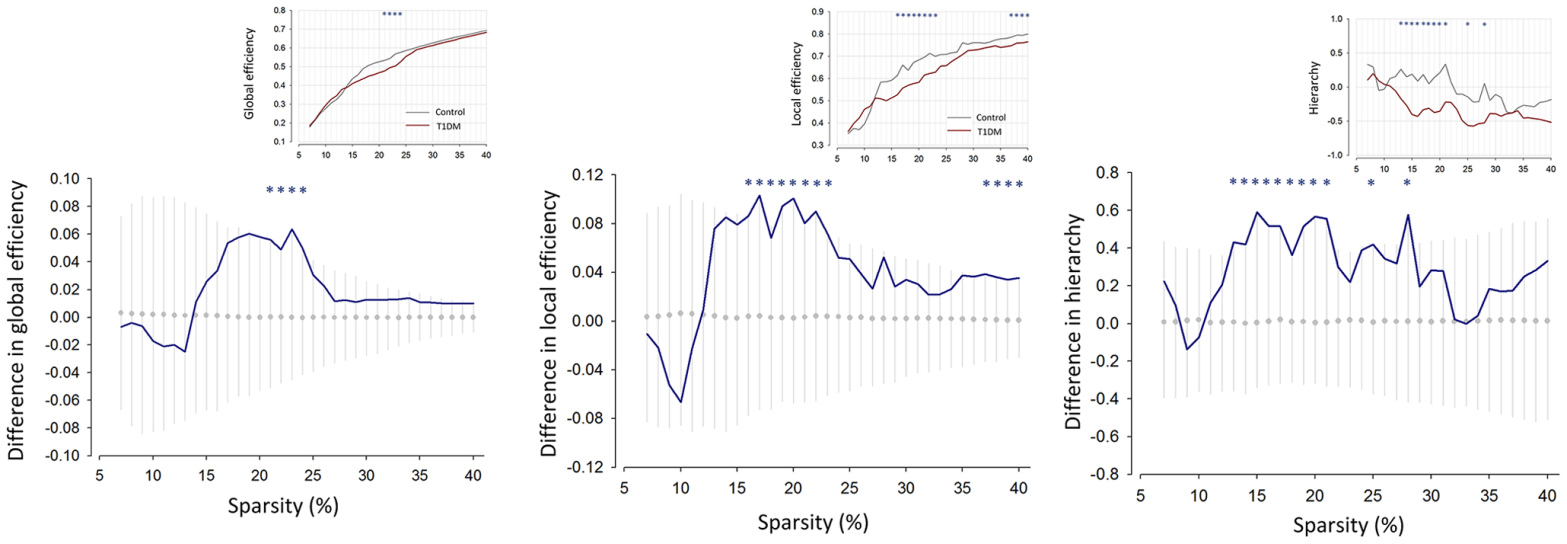
Between-group differences in global efficiency, local efficiency, and hierarchical organization of whole-brain structural networks. The graphs showed the differences in network parameters between the T1DM and control groups (blue line). The mean values and 95% of confidence interval of the null distribution of between-group differences obtained from 1000 permutation tests at each sparsity level were represented as gray circles and error bars, respectively. Asterisks indicate significant differences in parameters between the T1DM and control groups at *P*<0.05. Inset graphs show the global efficiency (*E_glob_)*, local efficiency (*E_loc_*), and hierarchical organization (β) of whole-brain structural networks in T1DM (red line) and control (gray line) subjects as functions of sparsity thresholds. Abbreviations: T1DM, type 1 diabetes mellitus.

We first assessed the global and local efficiencies of whole-brain structural networks, and compare the results between the T1DM and control groups. The global efficiency values (*E_glob_*) and the local efficiency (*E_loc_*) of whole-brain structural networks of T1DM patients were low at a wide range of sparsity levels relative to control subjects (inset graphs in Figure 1). Statistical analyses demonstrated that the T1DM group had significantly lower *E_glob_* than the control group in the sparsity range of 0.21≤*S*≤0.24. Statistically significant T1DM-related lower *E_loc_* values were also observed in sparsity range of 0.16≤*S*≤0.23 and 0.37≤*S*≤0.40 (Figure 1).

### Inter-network efficiencies between intrinsic structural sub -network systems

In order to examine whether the hierarchical cognitive control of the prefrontal cortex was altered in T1DM patients, we investigated inter-network efficiencies between structural network systems for strategic/executive control and other major brain functions including language, mnemonic/emotional processing, and sensorimotor function.

**Figure 2:**
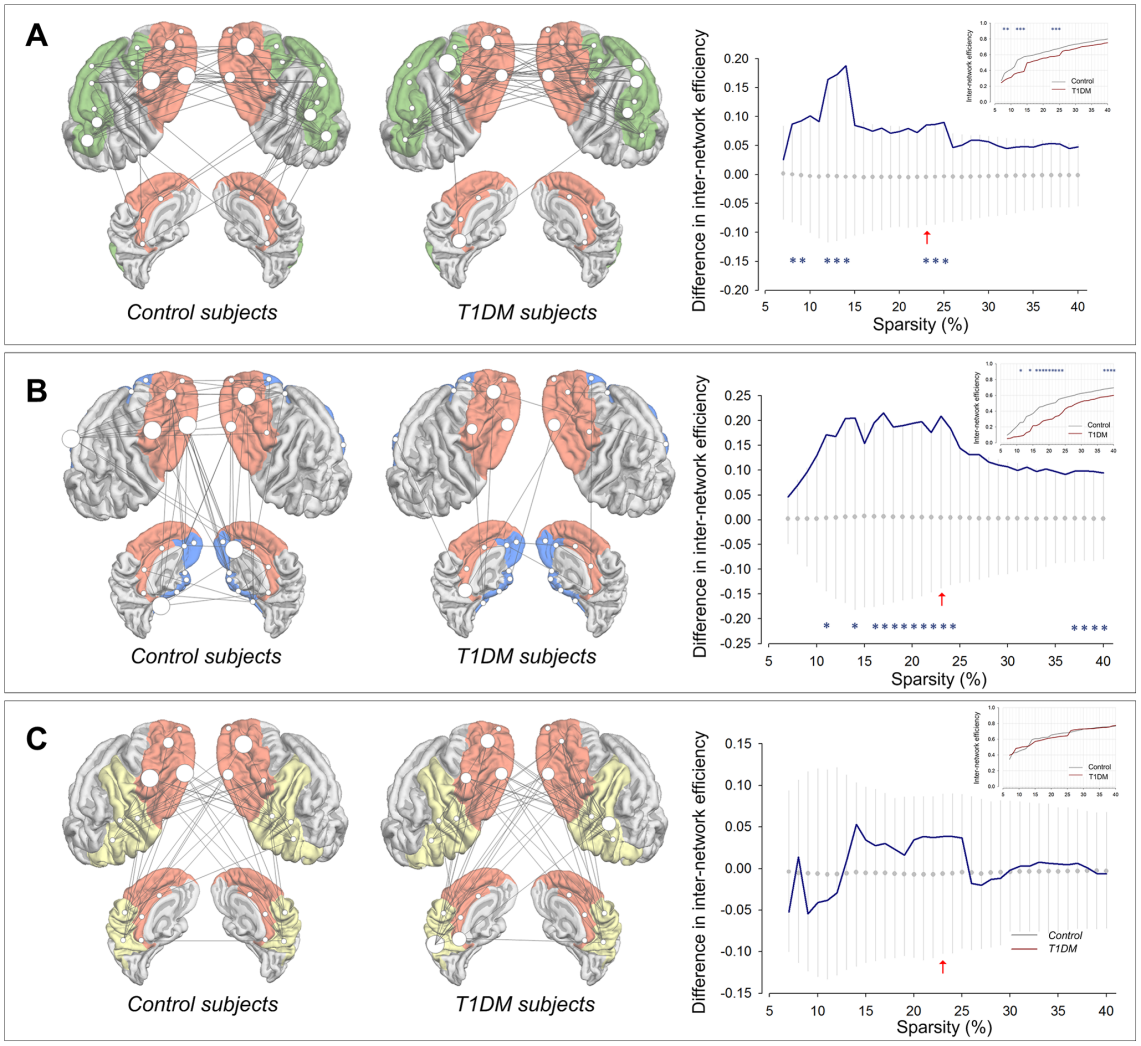
Connections and between-group differences in inter-network efficiencies. Connections of cortical structural network for strategic/executive control with other networks mediating language (A), mnemonic/emotional processing (B), and sensorimotor function (C) and between-group differences in inter-network efficiencies are presented in the left and right panels, respectively. Brain templates in figures demonstrate cortical parcellated regions for corresponding intrinsic cortical structural sub-network systems and inter-network connections at the sparsity threshold of 0.23. Red arrows in graphs indicate the sparsity of 0.23 that whole-brain structural networks of both control and T1DM groups included all 64 connected brain regions. Hub regions shown in Figure 3 are marked as larger white circles with the radius in proportion of the value of *B_i_*. The graphs showed the differences in average inverse shortest path length of submatrix for each corresponding intrinsic cortical structural network system between the T1DM and control groups (blue line). The mean values and 95% of confidence interval of the null distribution of between-group differences in parameters obtained from 1000 permutation tests at each sparsity level were represented as gray circles and error bars, respectively. Asterisks indicate significant differences in average inverse shortest path length between the T1DM and control groups at *P*<0.05. Inset graphs show average inverse shortest path length of submatrix representing inter-network efficiency, was plotted as a function of sparsity thresholds in T1DM (red line) and control (gray line) subjects. Abbreviations: T1DM, type 1 diabetes mellitus.

Average inverse shortest path length (*1/L*) between intrinsic structural sub-network systems was compared between groups. Statistical analyses showed that average inverse shortest path length between the strategic/executive control system and language system was lower in T1DM patients than in control subjects at 0.08≤*S*≤0.09, 0.12≤*S*≤0.14, and 0.23≤*S*≤0.25 (Figure 2A). T1DM patients also showed lower values of average inverse shortest path length between the strategic/executive control system and mnemonic/emotional processing system relative to control subjects in the sparsity range of 0.11, 0.14, 0.16≤*S*≤0.24, and 0.37≤*S*≤0.40 (Figure 2B). However, no between-group difference was observed in average inverse shortest path length between the strategic/executive control system and sensorimotor system (Figure 2C).

### Distributions of structural hubs and hierarchical organization of whole-brain structural networks

Sixty-four gyral-based parcellated regions were defined to generate a correlation matrix (Figure S1 in File S1). Graph theoretical properties of each parcellated cortical region including degree (*K_i_*), clustering coefficient (*C_i_*), and normalized betweenness-centrality (*B_i_*) values for both T1DM and control groups are demonstrated in Figure S2 in File S1.

**Figure 3:**
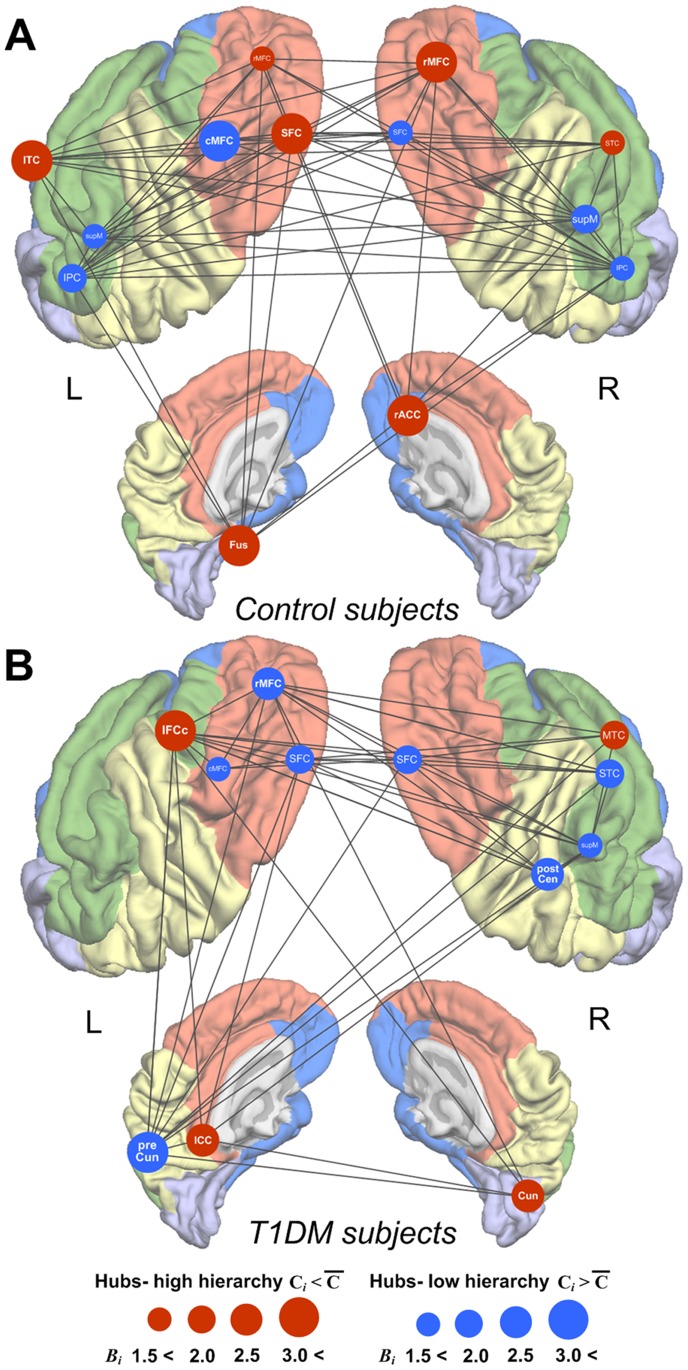
Location of hubs and inter-hub structural connections in whole-brain structural networks. Regions (brain templates of panels A for control and B T1DM subjects) in orange, green, blue, yellow, and light purple colors represent each intrinsic cortical structural sub-network system subserving strategic/executive control, language, mnemonic/emotional processing, sensorimotor, and visual functions, respectively. Figures depict hub regions and significant inter-hub structural connections of control (A) and T1DM (B) subjects at the sparsity threshold of 0.23. A given region was identified as a hub of whole-brain structural networks if its normalized betweenness-centrality (*B_i_*) was greater than 1.5 *and* its degree (*K_i_*) was above the network mean at the sparsity threshold of 0.23. Red circles denote the hub regions with low clustering (less than average clustering of whole-brain structural networks) indicating hubs at a higher position in the hierarchical organization. Blue circles denote the hub regions with high clustering (greater than average clustering of whole-brain structural networks) indicating hubs at a lower position in the hierarchical organization. The radius of circles is in proportion of the value of *B_i_* of the region at sparsity threshold of 0.23. Abbreviations: T1DM, type 1 diabetes mellitus; R, right; L, left; SFC, superior frontal cortex; rMFC, rostral middle frontal cortex; cMFC, caudal middle frontal cortex; ICC, isthmus cingulate cortex; IFCc, inferior frontal cortex- pars opercularis; IPC, inferior parietal cortex; supM, supramarginal cortex; STC, superior temporal cortex; MTC, middle temporal cortex; rACC, rostral anterior cingulate cortex; ITC, inferior temporal cortex; Fus, fusiform cortex; postCen, postcentral cortex; preCun, precuneus cortex; Cun, cuneus cortex.

As shown in Figure 3, the regional distribution of hubs and inter-hub structural connections in T1DM patients was considerably different from that in control subjects. The most prominent between-group differences were observed in the prefrontal and paralimbic regions that are known to be responsible for strategic/executive function and mnemonic/emotional processing, respectively. In particular, while healthy individuals had several hubs at a hierarchically higher level in the dorsolateral and paralimbic regions (red circles of Figure 3A), T1DM patients did not show this regional pattern of hierarchically high-level hubs in the same regions. The dorsolateral prefrontal hubs of T1DM patients were highly clustering, and thus had a hierarchically lower level (blue circles of Figure 3B). In addition, the degree and betweenness-centrality measures of the prefrontal hubs were reduced in the T1DM group as compared with the control group (Figures S2 and S3 in File S1).

Modular organization of structural cortical network in the T1DM and control groups was presented in Figure S4 in File S1. Similar findings for the group differences in regional distribution of nodes with higher inter-module connectivity were also observed in these modular analyses. Four connector hubs (defined as regions of *P_i_* >0.05 and *Z_i_* >1.0) [44], which were located on the right dorsolateral prefrontal cortex, the right anterior cingulate cortex, and the left temporal cortex, were identified in the structural cortical network of the control group (Figure S5 in File S2). However, the connector hubs in the T1DM group were mainly distributed in the right temporal cortex not in the prefrontal cortex (Figure S5 in File S2).

Furthermore, the β values, which indicate the level of hierarchy in whole-brain structural networks, were also found to be larger in control subjects than in T1DM patients in a range of the sparsity (0.13≤*S*≤0.21, 0.24, 0.28) suggesting a more hierarchical organization of networks in the control group (Figure 1).

## Discussion

Our data provide novel evidence of T1DM-related alterations in the topological organization of structural brain networks, which was assessed by using cortical thickness data from high-resolution T1-weighted MR imaging. We found that hierarchically high-level prefrontal hubs were relatively absent in T1DM patients, and that the levels of the hierarchical organization of whole-brain structural networks were also lower in T1DM patients than in control subjects. By utilizing the division of brain regions into segregated intrinsic sub-network systems with functional significance, we also found that the structural connections between the prefrontal regions exerting top-down cognitive controls and other regions subserving complex brain functions including language and mnemonic/emotional processing were likely to be less integrated in T1DM patients relative to control subjects.

Previous studies have reported that the normal human brain demonstrates significant levels of hierarchical organization, as it is characterized by optimal connections for top-down control with minimum wiring costs [40], [41], both in anatomical and functional networks [9], [15], [41]. Our finding that control subjects had hierarchically higher positioned hubs predominantly in the prefrontal regions is consistent with previous literature [15]. In light of the prefrontal regional deficits frequently observed in T1DM patients [45]–[50] along with the dorsolateral prefrontal thickness reductions in the present T1DM sample (Figure S6 in File S2) [24], loss of hierarchical organization in these regions could be anticipated in T1DM patients.

The absence of hierarchically organized hubs in key regions, such as the prefrontal regions, suggests that the contribution of these regions to the network efficiency may be reduced in T1DM patients. Our inter-network analysis results (Figure 2) corroborate this speculation. Along with the similar profile of global and local efficiencies of whole brain networks between the normal and T1DM brains, this finding also suggests that T1DM-related network disorganization, which would reflect inefficient connections between brain areas, may occur in a region-specific manner.

A recent functional study, which assessed resting-state brain networks in young adults with T1DM, has demonstrated similar findings to ours [51]. The connectivity of networks related to ventral attention, language, and fronto-parietal circuits was decreased in T1DM patients relative to control subjects. In contrast, the connectivity of sensorimotor and visual networks appeared to be preserved in T1DM patients.

Although sample characteristics (old age vs. young age) and measurements for network construction (white matter connectivity vs. cortical thickness) might be different from ours, it should be noted that the structural white matter network for the type 2 diabetes mellitus (T2DM) brain was also altered in terms of decreased global and local efficiency [52]. Furthermore, T2DM-related whole-brain white matter network abnormalities were associated with slowing of information process [52]. Future studies would be necessary to examine the neurocognitive as well as clinical relevance of T1DM-related cortical network disorganization for example, the altered top-down control role of the prefrontal cortex.

As T1DM often starts during childhood and adolescence [5], the current findings could be understood from a developmental perspective. Emerging evidence suggests that regions that subserve more primary functions including sensory or motor processing typically mature in infancy and early childhood [53]. However, brain regions responsible for more complex functions may require additional intrinsic and extrinsic inputs for maturation, and thereby mature at much later ages from childhood through adolescence [6], [8], [53]–[55]. In the context of this heterochronous developmental trajectory, T1DM and related metabolic insults may typically influence the brain regions predominantly implicated in complex cognitive functions rather than more primary sensorimotor functions. Moreover, childhood and adolescence is a critical period during which patients may be more vulnerable to diabetes-related metabolic disturbances than adulthood [5].

Considering a recent longitudinal observation suggesting minimal age-related brain volume loss in adolescents with T1DM [56], the current alterations in structural connections of the frontal cortex with other brain regions may reflect the effects of T1DM-related metabolic insult on the brain over a longer period of time beyond adolescence. For example, several cellular mechanisms including hyperglycemia-induced mitochondrial dysfunction and changes in synaptic plasticity [57], [58] could also have interactions with developmental reorganization of cortex in response to T1DM.

It is unclear why the structural connections subserving prefrontal controls on sensorimotor cortices were likely to be retained relative to those on other functions. Subclinical involvements of the retina or peripheral nerves in T1DM, which could occur even in the early stage of disease [59], [60], might precipitate the brain activities to compensate for the altered information conveyed by the peripheral nervous systems [51].

As suggested by previous diffusion tensor imaging studies that could provide anatomical information on the connections between brain regions, white matter connectivity in a wide range of brain areas may be affected in T1DM patients relative to control subjects [61]–[63]. Although findings of specific regional vulnerability of white matter to T1DM-related metabolic insult may be rather inconsistent, partly because of age span of study participants as well as implemented methodologies, studies demonstrated not only fronto-temporal but also parieto-occipital regional involvements related to T1DM [61]–[63]. The present with a sample of young adults with T1DM provides the descriptive findings of relatively preserved hubs in the parieto-occipital regions in T1DM. The functional relevance of regionally altered hierarchy of the T1DM brain should be further investigated in future studies using multimodal imaging analyses.

T1DM may lead to several neurobehavioral abnormalities, as the CNS manifestations of T1DM include impaired performances on several cognitive domains as well as a wide range of emotional problems such as depression and anxiety [5], [64]–[66]. An increasing number of neuroimaging studies suggest that the frontal and temporal brain regions may be the neuroanatomical correlates of these neurobehavioral abnormalities [24], [45]–[50], [67]–[69], in that these regions, which are known to have high insulin-receptor density and require high levels of cerebral energy, are particularly vulnerable to abnormal brain glucose metabolism [70]–[72].

The current findings suggest that altered inter-network connectivity between these key regions may play an important role in T1DM-associated neurobehavioral abnormalities, in addition to T1DM-related volume or thickness reductions with a similar regional pattern. In contrast, network efficiencies between brain regions implicated in the earliest developed functions such as sensorimotor function were preserved in T1DM patients.

One might assume that the metabolic load including history of glycemic control might affect the extent of alterations of structural cortical network in T1DM patients as shown in dose-dependent effects of hyperglycemia on neurochemical disturbances in the prefrontal cortex of T1DM patients [46]. Although our subgroup analyses using a non-parametric permutation test could not find the differences in global and local efficiencies of whole-brain structural cortical network between the good and poor glycemic control T1DM subgroups (Figure S7 in File S2) partly because of a relatively small sample size of each subgroup, the potential effects of disease load including glycemic control levels should be examined in the future studies with a sufficient sample size of T1DM. Furthermore, a longitudinal follow up of the T1DM sample to evaluate the progression of illness would also be important in providing valuable information on the dose-dependent relationship between T1DM-related clinical characteristics and alterations in structural cortical network.

Several limitations should be taken into account in interpreting the results. First, it remains to be determined whether the inter-regional correlation patterns of cortical thickness could reflect the alterations in intrinsic network systems for specific brain functions. However, growing evidence suggests that shared morphological organization may occur in functionally synchronous brain regions due to mutually trophic or neuroplastic influences [15], [19]. Recent structural imaging studies using diffusion tensor imaging also validated that the correlations of cortical thickness could indirectly indicate the anatomical connectivity of white matter tracts between regions [73]. For example, strong inter-regional correlations of cortical thickness were observed particularly where regions are axonally connected [73].

It should also be noted that anatomical connection matrices based on cortical thickness data that were used in the present study, were constructed by measuring inter-regional correlation coefficients over a group of subjects. In contrast to individual levels of connection matrices yielded from functional imaging data, we could not determine the network information at individual levels as well as the effects of inter-individual clinical variances on network organization. Furthermore, future studies using different cortical parcellation maps would be needed to replicate the current results based on Desikan-Killiany cortical atlas [35].

Although evidence based on histological and neuroimaging studies has indicated that cortical thickness may reflect composite estimations of size, density, and arrangement of cortical neurons, glia, or nerve fibers [74]–[76], a pending consensus on cellular substrates of changes in cortical thickness should also be considered in inferring the altered patterns of the anatomical connections matrix based on cortical thickness.

An Increasing body of literature has provided evidence that topological changes in brain networks may reflect disease progression or plastic changes in response to external stimuli [2]–[4]. Our data indicate that T1DM, occurring in childhood, may influence the normal development of topological organizations specifically responsible for higher complex functions such as strategic/executive control and mnemonic/emotional processing. Specifically, structural organization underlying top-down cognitive control of language, memory, and emotion by the prefrontal cortex may be affected in T1DM patients. These findings of T1DM-related network disorganization also provide a clue to the brain-based mechanisms of common long-term T1DM complications including cognitive decline or emotional disturbances.

## Supporting Information

File S1 **Supporting figures. Figure S1.** Cortical parcellation maps for construction of cortical structural network. The entire cerebral cortex was segmented into 64 cortical regions according to a prior surface parcellation based on the Desikan-Killiany Atlas for the construction of cortical structural network correlation matrix. Regions in orange, green, blue, yellow, and light purple colors represent each cortical structural sub-network exerting strategic/executive control, language, mnemonic/emotional processing, sensorimotor, and visual functions, respectively. Abbreviations: R, right; L, left; SFC, superior frontal cortex; rMFC, rostral middle frontal cortex; cMFC, caudal middle frontal cortex; IFCa, inferior frontal cortex- pars orbitalis; IFCb, inferior frontal cortex- pars triangularis; IFCc, inferior frontal cortex, pars opercularis; LOFC, lateral orbitofrontal cortex; MOFC, medial orbitofrontal cortex; preCen, precentral cortex; paraCen, paracentral cortex; Fpol, frontal pole; STC, superior temporal cortex; MTC, middle temporal cortex; ITC, inferior temporal cortex; TTC, transverse temporal cortex; Fus, fusiform cortex; Ento, entorhinal cortex; paraHi, parahippocampal cortex; Tpol, temporal pole; SPC, superior parietal cortex; IPC, inferior parietal cortex; supM, supramarginal cortex; postCen, postcentral cortex; preCun, precuneus cortex; rACC, rostral anterior cingulate cortex; cACC, caudal anterior cingulate cortex; PCC, posterior cingulate cortex; ICC, isthmus cingulate cortex; LOC, lateral occipital cortex; Cun, Cuneus; PeriCal, pericalcarine cortex; Lin, lingual cortex. **Figure S2.** Graph theoretical properties of parcellated cortical regions. Major brain functions subserved by parcellated regions are represented in distinct colors (orange, strategic/executive control; green, language function; blue, mnemonic/emotional processing; yellow, sensorimotor function; light purple, visual function, respectively). Abbreviations corresponding to the names of parcellated cortical regions are demonstrated in Figure S1. **A.** Comparison in the degree (*K_i_*) of each parcellated cortical region between control and T1DM subjects. The length of each bar indicates the degree of each parcellated cortical region. Degree is defined as the number of edges (connections) to other regions throughout the network at the sparsity threshold of 0.23. Regions were sorted by degree in descending order for each group. The values for degree of hierarchically high hub regions are presented as red bars while those of hierarchically low hub regions are presented as blue bars. Note that the high-degree regions of control subjects were likely to be at a hierarchically high level (low-clustering). In contrast, the high-degree regions of T1DM subjects were likely to be located at a hierarchically lower level (high-clustering). **B.** Comparison in the clustering coefficient (*C_i_*) of each parcellated cortical region between control and T1DM subjects. The length of each bar indicates the clustering coefficient of each parcellated cortical region. The clustering coefficient is defined as the ratio of the number of connections of that region with its nearest regions (neighbors) to the maximum number of possible connections in the network at the sparsity threshold of 0.23. Regions were sorted by clustering coefficient in descending order for each group. The values for degree of hierarchically high hub regions are presented as red bars while those of hierarchically low hub regions are presented as blue bars. **C.** Comparison in the normalized betweenness-centrality (*B_i_*) of each parcellated cortical region between control and T1DM subjects. The length of each bar indicates the normalized betweenness-centrality of each parcellated cortical region. Betweenness-centrality is defined as the number of shortest paths between any two regions that pass this specific region at the sparsity threshold of 0.23. Regions were sorted by normalized betweenness-centrality in descending order for each group. The values for degree of hierarchically high hub regions are presented as red bars while those of hierarchically low hub regions are presented as blue bars. Note that the regions with high centrality were likely to be at a hierarchically high level (low-clustering) in control subjects. In contrast, the regions with high centrality were likely to be located at a hierarchically lower level (high-clustering) in T1DM patients. **Figure S3.** Topological properties of hubs in whole-brain structural networks of control (A) and T1DM subjects (B). Bar graphs depict the degrees (*K_i_*) and clustering coefficients (*C_i_*) of each hub region in control (left) and T1DM subjects (right) at sparsity threshold of 0.23. Red dotted lines of bar graphs indicate the mean clustering coefficients of whole-brain network of control (*C_p_* = 0.55, left) and T1DM (*C_p_*  = 0.49, right) subjects. Abbreviations: T1DM, type 1 diabetes mellitus; R, right; L, left; SFC, superior frontal cortex; rMFC, rostral middle frontal cortex; cMFC, caudal middle frontal cortex; ICC, isthmus cingulate cortex; IFCc, inferior frontal cortex- pars opercularis; IPC, inferior parietal cortex; supM, supramarginal cortex; STC, superior temporal cortex; MTC, middle temporal cortex; rACC, rostral anterior cingulate cortex; ITC, inferior temporal cortex; Fus, fusiform cortex; postCen, postcentral cortex; preCun, precuneus cortex; Cun, cuneus cortex. **Figure S4.** Modular organization of the structural cortical network of the T1DM and control groups. The binarized matrix at a sparsity threshold of 0.23 for the control and T1DM groups were presented. Four modules were detected in the control group (left column). Module I contains 22 cortical regions including RSFC, RcMFC, LcMFC, RpreCen, LpreCen, RparaCen, LparaCen, RSPC, LSPC, LIPC, RsupraM, RpostCen, LpostCen, RpreCun, LpreCun, LcACC, RSTC, LSTC, RTCC, LEnto, LTPol, and RCun (yellow regions in the left column). Module II contains 22 cortical regions including RrMFC, LIFCc, RIFCb, RIFCa, RLOFC, RMOFC, LMOFC, RFpol, LFPol, RIPC, RrACC, LrACC, RcACC, RPCC, LPCC, RICC, LICC, RFus, LTTC, RparaHi, LparaHi, and RLOC (green regions in the left column). Module III contains 14 cortical regions including LSFC, LrMFC, RIFCc, LIFCb, LIFCa, LLOFC, LsupraM, RMTC, LMTC, RITC, LITC, LFus, REnto, and RTpol (blue regions in the left column). Module IV contains 6 cortical regions including LLOC, Lcun, RperiCal, LperiCal, RLin, and LLin (light yellow regions in the left column). The T1DM structural cortical network consists of 4 modules (right column). Module I contains 20 cortical regions including RSFC, LSFC, RrMFC, LrMFC, RcMFC, LcMFC, RIFCb, LIFCb, RpreCen, LpreCen, RparaCen, LparaCen, RSPC, LSPC, RIPC, LIPC, RsupraM, LsupraM, LpostCen, and RpreCun (yellow regions in the right column). Module II contains 20 cortical regions including LIFCc, LIFCa, RLOFC, LLOFC, RMOFC, LMOFC, RFpol, LFpol, RrACC, LrACC, RcACC, LcACC, RPCC, LPCC, RICC, LICC, RTPol, RparaHi, LparaHi, and RCun (green regions in the right column). Module III contains 17 cortical regions including RIFCc, RIFCa, LpreCun, RSTC, LSTC, RMTC, LMTC, RITC, LITC, RFus, LFus, RTTC, LTTC, REnto, LEnto, LTPol, and RLOC (blue regions in the right column). Module IV contains 7 cortical regions including RpostCen, LLOC, LCun, RperiCal, LperiCal, RLin, and LLin (light yellow regions in the right column). Note that the control group had more dorsolateral prefrontal regions of higher inter-module connectivity (*P_i_* >0.6)(red circles, LSFC, LrMFC, RrMFC) than the T1DM group (red circle. LrMFC). See also Figure S5. Abbreviations: T1DM, type 1 diabetes mellitus. Abbreviations corresponding to the names of parcellated cortical regions are demonstrated in Figure S1.
(DOC)

File S2 **Supporting figures. Figure S5.** Inter-module connectivity (participation coefficient, *P_i_*) and intra-module connectivity (z scores of within-module degree, *Z_i_*) of each cortical region for the control (left column) and T1DM (right column) groups. Regions with blue arrows indicate those with participation coefficients greater than 0.6 suggesting the strong inter-module connectivity. Regions with red arrows indicate those with z scores of within-module degree greater than 1. The node with *P_i_* >0.05 was defined as a connector node while that with *P_i_* ≤0.05 was defined as a provincial node (Meunier et al, 2009). The node with Z*_i_* >1 was defined as a hub (Meunier et al, 2009). According to this criteria, 4 connector hubs including RrMFC, RcMFC, RrACC, and LITC were identified in the structural cortical network of the control group. T1DM cortical network also had 4 connector hubs which were located on RrACC, RSTC, RMTC, and RFus. Abbreviations: T1DM, type 1 diabetes mellitus.Abbreviations corresponding to the names of parcellated cortical regions are demonstrated in Figure S1. **Figure S6.** Group difference in cortical thickness of each parcellated region between control and T1DM subjects. Group differences in cortical thickness of each parcellated region are represented as *z* scores using group mean thickness and standard deviation values of the control group (gray bars). Regions were sorted by the z scores of thickness difference in ascending order. A negative z score indicates the number of standard deviation units thinner than expected thickness of control subjects. A reduction in dorsolateral prefrontal cortical thickness (left superior frontal, left caudal middle frontal, and right superior cortices, regions with *z* scores less than −0.70) was observed in T1DM patients relative to control subjects. Major brain functions subserved by each parcellated regions are represented in separate colors (orange, strategic/executive control; green, language function; blue, mnemonic/emotional processing; yellow, sensorimotor function; light purple, visual function, respectively). Abbreviations corresponding to the name of parcellated cortical regions are demonstrated in Figure S1. **Figure S7.** Global efficiency and local efficiency of whole-brain structural networks in the T1DM subgroups according to lifetime glycemic control levels. A non-parametric permutation test with 1000 repetitions showed no statistically significant differences in mean values of *E_glob_* as well as local efficiency (*E_loc_*) across sparsity between the good and poor glycemic control T1DM groups. ^1^Lifetime average HbA1C level less than 7% (n = 18, mean lifetime average HbA1C = 6.55%). ^2^Lifetime average HbA1C level 7% or greater (n = 63, mean lifetime average HbA1C = 8.40%).
(DOC)
