# Supplementary material for: Network-Level Structural Abnormalities of Cerebral Cortex in Type 1 Diabetes Mellitus
Source: PLoS One. 2013 Aug 23;8(8):e71304. doi: 10.1371/journal.pone.0071304 (PMC3751935; doi:10.1371/journal.pone.0071304)
Supplement: File S1 — Supporting figures. Figure S1. Cortical parcellation maps for construction of cortical structural network. The entire cerebral cortex was segmented into 64 cortical regions according to a prior surface parcellation based on the Desikan-Killiany Atlas for the construction of cortical structural network correlation matrix. Regions in orange, green, blue, yellow, and light purple colors represent each cortical structural sub-network exerting strategic/executive control, language, mnemonic/emotional processing, sensorimotor, and visual functions, respectively. Abbreviations: R, right; L, left; SFC, superior frontal cortex; rMFC, rostral middle frontal cortex; cMFC, caudal middle frontal cortex; IFCa, inferior frontal cortex- pars orbitalis; IFCb, inferior frontal cortex- pars triangularis; IFCc, inferior frontal cortex, pars opercularis; LOFC, lateral orbitofrontal cortex; MOFC, medial orbitofrontal cortex; preCen, precentral cortex; paraCen, paracentral cortex; Fpol, frontal pole; STC, superior temporal cortex; MTC, middle temporal cortex; ITC, inferior temporal cortex; TTC, transverse temporal cortex; Fus, fusiform cortex; Ento, entorhinal cortex; paraHi, parahippocampal cortex; Tpol, temporal pole; SPC, superior parietal cortex; IPC, inferior parietal cortex; supM, supramarginal cortex; postCen, postcentral cortex; preCun, precuneus cortex; rACC, rostral anterior cingulate cortex; cACC, caudal anterior cingulate cortex; PCC, posterior cingulate cortex; ICC, isthmus cingulate cortex; LOC, lateral occipital cortex; Cun, Cuneus; PeriCal, pericalcarine cortex; Lin, lingual cortex. Figure S2. Graph theoretical properties of parcellated cortical regions. Major brain functions subserved by parcellated regions are represented in distinct colors (orange, strategic/executive control; green, language function; blue, mnemonic/emotional processing; yellow, sensorimotor function; light purple, visual function, respectively). Abbreviations corresponding to the names of parcellated [file pone.0071304.s001.doc]

**Network-Level Structural Abnormalities of Cerebral Cortex in Type 1 Diabetes Mellitus**

***Supporting Information (File S1)***

**Figure S1.** Cortical parcellation maps for construction of cortical structural network.

**Figure S2.** Graph theoretical properties of parcellated cortical regions.

**Figure S3.** Topological properties of hubs in whole-brain structural networks of control (A) and T1DM subjects (B).

**Figure S4.** Modular organization of the structural cortical network of the T1DM and control groups.

|  |
| --- |

**Figure S1.** Cortical parcellation maps for construction of cortical structural network.

The entire cerebral cortex was segmented into 64 cortical regions according to a prior surface parcellation based on the Desikan-Killiany Atlas for the construction of cortical structural network correlation matrix. Regions in orange, green, blue, yellow, and light purple colors represent each cortical structural sub-network exerting strategic/executive control, language, mnemonic/emotional processing, sensorimotor, and visual functions, respectively.

Abbreviations: R, right; L, left; SFC, superior frontal cortex; rMFC, rostral middle frontal cortex; cMFC, caudal middle frontal cortex; IFCa, inferior frontal cortex- pars orbitalis; IFCb, inferior frontal cortex- pars triangularis; IFCc, inferior frontal cortex, pars opercularis; LOFC, lateral orbitofrontal cortex; MOFC, medial orbitofrontal cortex; preCen, precentral cortex; paraCen, paracentral cortex; Fpol, frontal pole; STC, superior temporal cortex; MTC, middle temporal cortex; ITC, inferior temporal cortex; TTC, transverse temporal cortex; Fus, fusiform cortex; Ento, entorhinal cortex; paraHi, parahippocampal cortex; Tpol, temporal pole; SPC, superior parietal cortex; IPC, inferior parietal cortex; supM, supramarginal cortex; postCen, postcentral cortex; preCun, precuneus cortex; rACC, rostral anterior cingulate cortex; cACC, caudal anterior cingulate cortex; PCC, posterior cingulate cortex; ICC, isthmus cingulate cortex; LOC, lateral occipital cortex; Cun, Cuneus; PeriCal, pericalcarine cortex; Lin, lingual cortex.

| **Figure S2.** Graph theoretical properties of parcellated cortical regions.  Major brain functions subserved by parcellated regions are represented in distinct colors (orange, strategic/executive control; green, language function; blue, mnemonic/emotional processing; yellow, sensorimotor function; light purple, visual function, respectively). Abbreviations corresponding to the names of parcellated cortical regions are demonstrated in Figure S1. |
| --- |

| **A.** Comparison in the degree (*K_i_*) of each parcellated cortical region between control and T1DM subjects |
| --- |
|  |
| The length of each bar indicates the degree of each parcellated cortical region. Degree is defined as the number of edges (connections) to other regions throughout the network at the sparsity threshold of 0.23. Regions were sorted by degree in descending order for each group. The values for degree of hierarchically high hub regions are presented as red bars while those of hierarchically low hub regions are presented as blue bars. Note that the high-degree regions of control subjects were likely to be at a hierarchically high level (low-clustering). In contrast, the high-degree regions of T1DM subjects were likely to be located at a hierarchically lower level (high-clustering). |

| **B.** Comparison in the clustering coefficient (*C_i_*) of each parcellated cortical region between control and T1DM subjects. |
| --- |
|  |
| The length of each bar indicates the clustering coefficient of each parcellated cortical region. The clustering coefficient is defined as the ratio of the number of connections of that region with its nearest regions (neighbors) to the maximum number of possible connections in the network at the sparsity threshold of 0.23. Regions were sorted by clustering coefficient in descending order for each group. The values for degree of hierarchically high hub regions are presented as red bars while those of hierarchically low hub regions are presented as blue bars. |

| **C.** Comparison in the normalized betweenness-centrality (*B_i_*) of each parcellated cortical region between control and T1DM subjects. |
| --- |
|  |
| The length of each bar indicates the normalized betweenness-centrality of each parcellated cortical region. Betweenness-centrality is defined as the number of shortest paths between any two regions that pass this specific region at the sparsity threshold of 0.23. Regions were sorted by normalized betweenness-centrality in descending order for each group. The values for degree of hierarchically high hub regions are presented as red bars while those of hierarchically low hub regions are presented as blue bars. Note that the regions with high centrality were likely to be at a hierarchically high level (low-clustering) in control subjects. In contrast, the regions with high centrality were likely to be located at a hierarchically lower level (high-clustering) in T1DM patients. |

|  |
| --- |

**Figure S3.** Topological properties of hubs in whole-brain structural networks of control (A) and T1DM subjects (B).

Bar graphs depict the degrees (*K_i_*) and clustering coefficients (*C_i_*) of each hub region in control (left) and T1DM subjects (right) at sparsity threshold of 0.23. Red dotted lines of bar graphs indicate the mean clustering coefficients of whole-brain network of control (*C_p_*=0.55*,* left) and T1DM (*C_p_* =0.49*,* right) subjects.

Abbreviations: T1DM, type 1 diabetes mellitus; R, right; L, left; SFC, superior frontal cortex; rMFC, rostral middle frontal cortex; cMFC, caudal middle frontal cortex; ICC, isthmus cingulate cortex; IFCc, inferior frontal cortex- pars opercularis; IPC, inferior parietal cortex; supM, supramarginal cortex; STC, superior temporal cortex; MTC, middle temporal cortex; rACC, rostral anterior cingulate cortex; ITC, inferior temporal cortex; Fus, fusiform cortex; postCen, postcentral cortex; preCun, precuneus cortex; Cun, cuneus cortex.

| 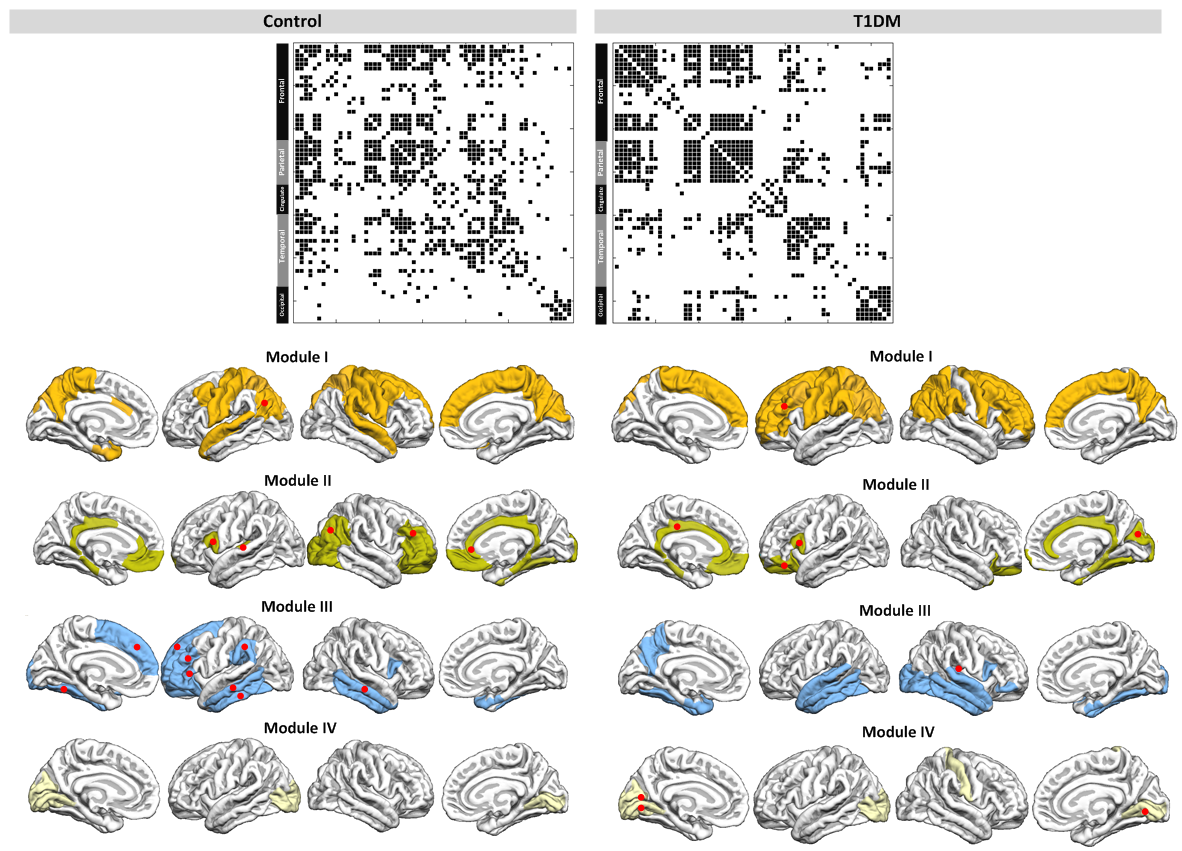 |
| --- |

**Figure. S4.** Modular organization of the structural cortical network of the T1DM and control groups.

The binarized matrix at a sparsity threshold of 0.23 for the control and T1DM groups were presented.

Four modules were detected in the control group (left column). Module I contains 22 cortical regions including RSFC, RcMFC, LcMFC, RpreCen, LpreCen, RparaCen, LparaCen, RSPC, LSPC, LIPC, RsupraM, RpostCen, LpostCen, RpreCun, LpreCun, LcACC, RSTC, LSTC, RTCC, LEnto, LTPol, and RCun (yellow regions in the left column). Module II contains 22 cortical regions including RrMFC, LIFCc, RIFCb, RIFCa, RLOFC, RMOFC, LMOFC, RFpol,LFPol, RIPC, RrACC, LrACC, RcACC, RPCC, LPCC, RICC, LICC, RFus, LTTC, RparaHi, LparaHi, and RLOC (green regions in the left column). Module III contains 14 cortical regions including LSFC, LrMFC, RIFCc, LIFCb, LIFCa, LLOFC, LsupraM, RMTC, LMTC, RITC, LITC, LFus, REnto, and RTpol (blue regions in the left column). Module IV contains 6 cortical regions including LLOC, Lcun, RperiCal, LperiCal, RLin, and LLin (light yellow regions in the left column).

The T1DM structural cortical network consists of 4 modules (right column). Module I contains 20 cortical regions including RSFC, LSFC, RrMFC, LrMFC, RcMFC, LcMFC, RIFCb, LIFCb, RpreCen, LpreCen, RparaCen, LparaCen, RSPC, LSPC, RIPC, LIPC, RsupraM, LsupraM, LpostCen, and RpreCun (yellow regions in the right column). Module II contains 20 cortical regions including LIFCc, LIFCa, RLOFC, LLOFC, RMOFC, LMOFC, RFpol, LFpol, RrACC, LrACC, RcACC, LcACC, RPCC, LPCC, RICC, LICC, RTPol, RparaHi, LparaHi, and RCun (green regions in the right column). Module III contains 17 cortical regions including RIFCc, RIFCa, LpreCun, RSTC, LSTC, RMTC, LMTC, RITC, LITC, RFus, LFus, RTTC, LTTC, REnto, LEnto, LTPol, and RLOC (blue regions in the right column). Module IV contains 7 cortical regions including RpostCen, LLOC, LCun, RperiCal, LperiCal, RLin and LLin (light yellow regions in the right column).

Note that the control group had more dorsolateral prefrontal regions of higher inter-module connectivity (*P_i_* > 0.6)(red circles, LSFC, LrMFC, RrMFC) than the T1DM group (red circle. LrMFC). See also Figure S5.

Abbreviations: T1DM, type 1 diabetes mellitus. Abbreviations corresponding to the names of parcellated cortical regions are demonstrated in Figure S1.
