# Supplementary material for: Network-Level Structural Abnormalities of Cerebral Cortex in Type 1 Diabetes Mellitus
Source: PLoS One. 2013 Aug 23;8(8):e71304. doi: 10.1371/journal.pone.0071304 (PMC3751935; doi:10.1371/journal.pone.0071304)
Supplement: File S2 — Supporting figures. Figure S5. Inter-module connectivity (participation coefficient, Pi) and intra-module connectivity (z scores of within-module degree, Zi) of each cortical region for the control (left column) and T1DM (right column) groups. Regions with blue arrows indicate those with participation coefficients greater than 0.6 suggesting the strong inter-module connectivity. Regions with red arrows indicate those with z scores of within-module degree greater than 1. The node with Pi >0.05 was defined as a connector node while that with Pi ≤0.05 was defined as a provincial node (Meunier et al, 2009). The node with Zi >1 was defined as a hub (Meunier et al, 2009). According to this criteria, 4 connector hubs including RrMFC, RcMFC, RrACC, and LITC were identified in the structural cortical network of the control group. T1DM cortical network also had 4 connector hubs which were located on RrACC, RSTC, RMTC, and RFus. Abbreviations: T1DM, type 1 diabetes mellitus.Abbreviations corresponding to the names of parcellated cortical regions are demonstrated in Figure S1. Figure S6. Group difference in cortical thickness of each parcellated region between control and T1DM subjects. Group differences in cortical thickness of each parcellated region are represented as z scores using group mean thickness and standard deviation values of the control group (gray bars). Regions were sorted by the z scores of thickness difference in ascending order. A negative z score indicates the number of standard deviation units thinner than expected thickness of control subjects. A reduction in dorsolateral prefrontal cortical thickness (left superior frontal, left caudal middle frontal, and right superior cortices, regions with z scores less than −0.70) was observed in T1DM patients relative to control subjects. Major brain functions subserved by each parcellated regions are represented in separate colors (orange, strategic/executive control; green, language function; blue, mnemonic/emotion [file pone.0071304.s002.doc]

**Network-Level Structural Abnormalities of Cerebral Cortex in Type 1 Diabetes Mellitus**

***Supporting Information (File S2)***

**Figure S5.** Inter-module connectivity (participation coefficient, *P_i_*) and intra-module connectivity (z scores of within-module degree, *Z_i_*) of each cortical region for the control (left column) and T1DM (right column) groups.

**Figure S6.** Group difference in cortical thickness of each parcellated region between control and T1DM subjects.

**Figure S7.** Global efficiency and local efficiency of whole-brain structural networks in the T1DM subgroups according to lifetime glycemic control levels.

|  |
| --- |

**Figure S5.** Inter-module connectivity (participation coefficient, *P_i_*) and intra-module connectivity (z scores of within-module degree, *Z_i_*) of each cortical region for the control (left column) and T1DM (right column) groups.

Regions with blue arrows indicate those with participation coefficients greater than 0.6 suggesting the strong inter-module connectivity. Regions with red arrows indicate those with z scores of within-module degree greater than 1.

The node with *P_i_* > 0.05 was defined as a connector node while that with *P_i_* ≤ 0.05 was defined as a provincial node (Meunier et al, 2009). The node with Z*_i_* > 1 was defined as a hub (Meunier et al, 2009). According to this criteria, 4 connector hubs including RrMFC, RcMFC,RrACC, and LITC were identified in the structural cortical network of the control group. T1DM cortical network also had 4 connector hubs which were located on RrACC, RSTC, RMTC, and RFus.

Abbreviations: T1DM, type 1 diabetes mellitus.Abbreviations corresponding to the names of parcellated cortical regions are demonstrated in Figure S1.

|  |
| --- |

**Figure S6.** Group difference in cortical thickness of each parcellated region between control and T1DM subjects.

Group differences in cortical thickness of each parcellated region are represented as *z* scores using group mean thickness and standard deviation values of the control group (gray bars). Regions were sorted by the z scores of thickness difference in ascending order. A negative z score indicates the number of standard deviation units thinner than expected thickness of control subjects. A reduction in dorsolateral prefrontal cortical thickness (left superior frontal, left caudal middle frontal, and right superior cortices, regions with *z* scores less than -0.70) was observed in T1DM patients relative to control subjects.

Major brain functions subserved by each parcellated regions are represented in separate colors (orange, strategic/executive control; green, language function; blue, mnemonic/emotional processing; yellow, sensorimotor function; light purple, visual function, respectively).

Abbreviations corresponding to the name of parcellated cortical regions are demonstrated in Figure S1.

|  |
| --- |

**Figure S7.** Global efficiency and local efficiency of whole-brain structural networks in the T1DM subgroups according to lifetime glycemic control levels.

A non-parametric permutation test with 1000 repetitions showed no statistically significant differences in mean values of *E_glob_* as well as local efficiency (*E_loc_*) across sparsity between the good and poor glycemic control T1DM groups.

^1^ Lifetime average HbA1C level less than 7 % (n =18, mean lifetime average HbA1C = 6.55%).

^2^ Lifetime average HbA1C level 7 % or greater (n = 63, mean lifetime average HbA1C = 8.40%).
